# Carbohydrates, Starch, Total Sugar, Fiber Intakes and Food Sources in Spanish Children Aged One to <10 Years—Results from the EsNuPI Study †

**DOI:** 10.3390/nu12103171

**Published:** 2020-10-16

**Authors:** Maria de Lourdes Samaniego-Vaesken, Teresa Partearroyo, Teresa Valero, Paula Rodriguez, María José Soto-Méndez, Ángela Hernández-Ruiz, Federico Lara Villoslada, Rosaura Leis, Emilio Martínez de Victoria, José Manuel Moreno, Rosa M. Ortega, María Dolores Ruiz-López, Ángel Gil, Gregorio Varela-Moreiras

**Affiliations:** 1 Departamento de Ciencias Farmacéuticas y de la Salud, Facultad de Farmacia, Universidad San Pablo-CEU, CEU Universities, Urbanización Montepríncipe, Alcorcón, 28925 Madrid, Spain; l.samaniego@ceu.es (M.d.L.S.-V.); t.partearroyo@ceu.es (T.P.); 2 Spanish Nutrition Foundation (FEN), c/General Álvarez de Castro 20, 1ªpta, 28010 Madrid, Spain; tvalero@fen.org.es (T.V.); prodriguez@fen.org.es (P.R.); 3 Iberoamerican Nutrition Foundation (FINUT), Av. del Conocimiento 12, 3ª pta, Armilla, 18016 Granada, Spain; msoto@finut.org (M.J.S.-M.); ahernandez@finut.org (Á.H.-R.); emiliom@ugr.es (E.M.d.V.); mdruiz@ugr.es (M.D.R.-L.); agil@ugr.es (Á.G.); 4 Instituto de Nutrición Puleva, Camino de Purchil 66, 18004 Granada, Spain; federico.lara@lactalis.es; 5 Department of Pediatrics, Unit of Pediatric Gastroenterology, Hepatology and Nutrition, University Clinical Hospital of Santiago, IDIS, ISCIII, University of Santiago de Compostela, 15700 Santiago de Compostela, Spain; mariarosaura.leis@usc.es; 6 CIBEROBN (Physiopathology of Obesity and Nutrition CB12/03/30038), Instituto de Salud Carlos III (ISCIII), 28029 Madrid, Spain; 7 Department of Physiology, Faculty of Pharmacy, Campus de Cartuja, University of Granada, s/n, 18071 Granada, Spain; 8 Pediatric Department, Calle Marquesado de Sta. Marta 1, University of Navarra Clinic, 28027 Madrid, Spain; jmoreno@unav.es; 9 Department of Nutrition and Food Science, Faculty of Pharmacy, Complutense University of Madrid, Plaza Ramón y Cajal s/n, 28040 Madrid, Spain; rortega@ucm.es; 10 Department of Nutrition and Food Science, Faculty of Pharmacy, Campus de Cartuja, s.n, University of Granada, 18071 Granada, Spain; 11 Institute of Nutrition and Food Technology “José Mataix,” Biomedical Research Center, University of Granada, Parque Tecnológico de la Salud, Avenida del Conocimiento s/n, Armilla, 18100 Granada, Spain; 12 Department of Biochemistry and Molecular Biology II, University of Granada, Campus de Cartuja, s.n, 18071 Granada, Spain

**Keywords:** carbohydrate intakes, EsNuPI study, pediatrics, Spanish children, feeding behavior, dietary habits, nutrition assessment, pediatric nutrition, total sugar

## Abstract

Diet quality is a modifiable factor that may contribute to the onset of diet-related chronic diseases. Currently, in Spain there are no studies that examine the intakes and sources for total carbohydrates, starch, total sugar, and fiber by both children consuming all kind of milks and children regularly consuming adapted milk formulas. Our goal was to evaluate the contribution of different food groups to total carbohydrates, starch, total sugar, and fiber consumption within the EsNuPI study participants by assessing their usual intakes by applying two 24 h dietary recalls that were completed by 1448 children (1 to <10 years) divided into two cohorts: one Spanish Reference Cohort (SRS) of the general population (*n* = 707) and another cohort which included children consuming adapted milks including follow-on milk, toddler’s or growing up milk, fortified and enriched milks, here called Adapted Milk Consumers Cohort” (AMS) (*n* = 741). Estimation of the usual intake showed that nutrient intake increased with age for all nutrients except for fiber. The percentage of children by age and gender who met the reference intake (RI) range for total carbohydrates, was in all groups more than 50% of individuals, except for girls aged 6 to <10 years from the reference cohort in which only 46.9% complied the RI. Median fiber intake, both in the SRS and the AMS, was well below the adequate intake (AI) for children between 3 and 10 years. Main total carbohydrates sources were cereals, followed by milk and dairy products, fruits, bakery and pastry, vegetables and sugars and sweets. The highest contributors to starch intakes were cereals, bakery and pastry, vegetables, and fruits. Major sources of total sugar intakes were milk and dairy products, fruits, bakery and pastry, sugars and sweets, vegetables, and cereals. Nonetheless, milk and dairy products, and fruits, mainly provided lactose and fructose, respectively, which are not considered free sugars. Higher contribution to fiber intakes was provided by fruits, cereals, vegetables and bakery and pastry. There were no significant differences in relation with the total sugar intake according to the body mass index (BMI) between SRS and AMS. The present study suggests a high proportion of children had total carbohydrates intakes in line with recommendations by public health authorities, but still a significant number presented insufficient total carbohydrate and fiber intakes, while total sugar consumption was high, with no major differences between SRS and AMS cohorts.

## 1. Introduction

Diet quality is a modifiable factor that may contribute to the onset of diet-related chronic diseases such as obesity, cardiovascular disease, type 2 diabetes, and some cancers [1]. In fact, the adoption of healthy eating patterns from an early age is a fundamental primary prevention tool for fighting these diseases. Childhood comprises a period of both vulnerability and opportunity [2], being specially significant for the implementation of adequate dietary habits that provide sufficient energy and nutrient intakes which, in turn, will be determinant of an optimal adult health status [2,3]. In this regard, the World Health Organization (WHO) acknowledged that child overweight and obesity is one of the most challenging public health issues faced over the 21st century and that its prevention should be a high priority for policy makers [4]. According to the latest National Health Survey results, childhood overweight and obesity in Spain accounts for almost 30% of the population [5]; moreover, data from “*The Heavy Burden of Obesity*” report published in 2019 [6] determined that Spain is in the fourth place amongst the European Union (EU), in the prevalence of overweight and obesity for individuals 5 to 19 years.

Dietary patterns from Spanish children have been previously described by a number of studies that revealed that the average diet needed to be improved from a macronutrient profile perspective, as it was high in protein and total fats and lower in carbohydrates, namely complex. According to worldwide health authorities, carbohydrates are the macronutrient category that should comprise the main contribution to total energy intakes (TEI) within our diets [7]. However, an important proportion of the Spanish population, specifically children, are not complying with this recommendation [8]. The National Dietary Survey on the Child and Adolescent Population project in Spain (ENALIA study, 2013–2014), which assessed the nutritional profile of Spanish individuals aged 6 months to 17 years (*n* = 1862), found that younger children needed to decrease their fat and protein intakes and increase carbohydrates [9]. Median values for carbohydrates were 46.8% of total energy (41.2–52.3%, P10–P90) and the usual median proportion of energy intake from carbohydrates was higher for infants from 6 to 12 months than for the other age groups. The ANIBES study (“Anthropometric data, macronutrients and micronutrients intake, practice of physical activity, socioeconomic data and lifestyles in Spain”), which also studied a representative cohort from the Spanish population in 2013, only included children aged 9 to 12 years, but found a similar pattern in macronutrient distribution as protein contributed to 16% of TEI, lipids to 38.9% and carbohydrates to 43.8% in this age group [10].

Starch, a complex carbohydrate, is the main glucose source from which our cells obtain energy and, therefore, dietary guidelines have always placed food groups containing it in the basis of our dietary requirements [7,11,12]. These food groups include cereals and derivatives (flour, bread, pasta, corn, rice, etc.), potatoes, legumes, tree nuts and some vegetables. On the other hand, simple carbohydrates (sucrose, fructose, lactose, etc.), also regarded as “sugars” are not deemed essential to our survival but provide sweet taste which is one of the major drivers of food acceptability and preference. A comprehensive review with data from 11 surveys that included Spain, assessed the total and added sugar intakes and its dietary sources in Europe, finding that relative intakes were excessive, namely amongst children [13]. The WHO has warned that excessive sugar intakes, mainly of free and added sugars, across individuals of all ages might be related to excess weight and other non-communicable diseases (NCDs) globally (i.e., insulin sensitivity, type 2-diabetes, cardiovascular risk factors, and dental caries) and recognizes it is a key public health issue [3].

In 2010, the European Food Safety Authority (EFSA) issued its Scientific Opinion on Dietary Reference Values (DRV) for carbohydrates and dietary fiber [7]. It comprises the recommendation for a daily intake of 45–60% of TEI as total carbohydrates but specify that there are insufficient data to set an upper limit for added sugar intakes. Nutritionally, “dietary fiber”, may be defined as a group of compounds found in plant-based foods, that comprise non-digestible carbohydrates plus lignin, and that may reach the large intestine. This group includes non-starch polysaccharides such as cellulose, hemicelluloses, pectins, hydrocolloids (i.e., gums, mucilages, β-glucans), resistant oligosaccharides, fructo-oligosaccharides (FOS), galactooligosaccharides (GOS) and resistant starch [7]. According to EFSA, average dietary fiber intakes in European countries varies from 10 to 20 g per day in young children (<10 to 12 years) [7]. Adequate intakes (AI) for dietary fiber have been established by EFSA based on the available evidence of their beneficial role in bowel function. It is considered that daily intakes of 25 g is suitable for normal laxation in adults [7]. AI for dietary fiber for children should be based on that for adults with appropriate adjustment for energy intake. A fiber intake of 2 g per MJ is considered adequate for normal laxation in children from the age of one year. AI established by EFSA recommend a daily fiber intake of 10 g for children 1 to 3 years, 14 g for those 4 to 6 years and 16 g for age range of 7 to 10 years [7]. Amongst the adult population, there is mounting evidence of the health benefits associated with diets rich in fiber-containing foods (>25 g/day) including reduced risk of coronary heart disease and type 2 diabetes, some types of cancer (i.e., colorectal), satiety and weight management [7]. The European Society for Pediatric Gastroenterology Hepatology and Nutrition (ESPGHAN) recommends that by school age a balanced diet should provide at least 10 g per day of dietary fiber, and that the intake should then progressively increase to reach the recommended level for adults during adolescence [14]. National dietary surveys showed globally that fiber intake from children is insufficient. In the ENALIA study, average usual fiber intakes ranged between 9.2 ± 3.4 g/day for children 6–12 months, to 18.4 ± 3.8 g/day for those aged 9–13 years [9]. In the ANIBES study, mean daily fiber intakes amongst children aged 9 to 12 years were 11.8 ± 4.3 g, slightly higher in girls than in boys but lower than values found by ENALIA. The dietary fiber sources in the current Spanish diet was found in descending order to be mainly from grains, vegetables, fruits, and pulses [8].

The necessity of the present study rises from the need to evaluate updated data on children’s dietary intakes. Data were compiled between 2018–2019 and to our knowledge, this is the latest study available on this population group (1–9 years old), including not only the evaluation of dietary intake but also physical activity and socioeconomical factors. In the present study we aimed to (1) determine the total carbohydrate, starch, total sugar, and fiber intakes, (2) to describe their main food sources, and (3) to analyze the differences between two cohorts, one representative of the one to <10 years of urban Spanish children consuming all kinds of milk, Spanish Reference Cohort (SRS) and the other, Adapted Milk Consumers Cohort (AMS) frequently consuming adapted milk formulas (follow-on milk, toddler’s or growing-up milk, fortified and enriched milks), who participated in the EsNuPI study (from the Spanish “*Estudio Nutricional en Población Infantil Española*” or “Nutritional Study in Spanish Pediatric Population”). This is the first study in our country in which dietary patterns and food sources are assessed and compared between two different types of milk consumer group. It provides the most recent update on dietary intakes from a population group which is critical for prevention of diet related diseases and, on the other hand, it provides new and relevant information about differences between children who consume adapted milks and those who doesn’t, a topic which lacks of scientific information despite the frequent controversy and misinformation.

## 2. Materials and Methods

### 2.1. Study Design and Sample

**Figure 1:**
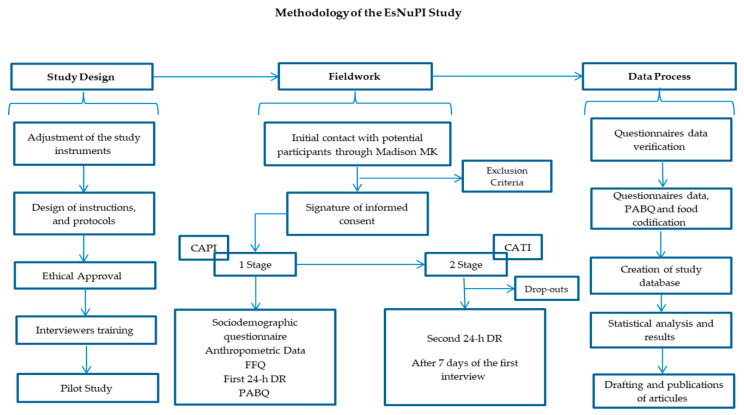
Design and methodology of the EsNuPI study. Taken from Madrigal et al., 2019 [15]. CAPI, Computer-Assisted Personal Interviewing; FFQ, food frequency questionnaire; 24-h DR, 24-h dietary recall; PABQ, physical activity and sedentary behaviors questionnaire; CATI, Computer-Assisted Telephone Interviewing.

The EsNuPI study is a prospective, cross-sectional, observational study, that took place from October 2018 to January 2019 and provided the data evaluated in the present work. The complete design, protocol and methodology have been previously published [15] and a schematic overview is provided in Figure 1. The EsNuPI study assessed the dietary patterns and nutrient intake, in addition to physical activity and sedentary behaviors of Spanish children, living in urban areas with >50,000 inhabitants, from nine regions as established by Nielsen Spanish areas. Two sub-cohorts were included from the Spanish population aged one to <10 years old, one comprising the urban non-vegan (Spanish Reference Cohort, SRS) individuals who consumed all kinds of milk in the last 12 months and another named “Adapted Milk Consumers Cohort, AMS” of non-vegan subjects also living in urban areas and consuming adapted milks over the last 12 months. Within the “adapted milks” denomination, infant formula, follow-on milk formula, toddler’s milk formula (also called “young children milk formula” and in Spain “growing up” milk formula) and fortified and enriched milk formula (e.g., docosahexaenoic acid (DHA), calcium, vitamin D, iron) were included. The initially estimated sample comprised 1500 individuals, and the sample errors were ±2.52% and ±2.59%, respectively, for a 95.5% confidence level and estimation of equally probable categories (*p* = q = 50%), considering a universe of 2,205,646 children. The selected population comprised 1514 children (*n* = 742 SRS; *n* = 772 AMS) who completed a face-to-face interview providing sociodemographic information, answering a quantitative food frequency questionnaire (FFQ), a physical activity and sedentary behaviors questionnaire (PABQ) and the first 24-h dietary recall (24-h DR). An additional 24-h DR was completed by telephone after seven days. Finally, 1448 individuals completed the study by answering the telephone survey, which accounted for a response rate of 95.6%. The EsNuPI study was performed in accordance with the declaration of Helsinki and was approved by the University of Granada ethical committee (No. 659/CEIH/2018) and registered in ClinicalTrials.gov (Unique Protocol ID: FF01/2019).

### 2.2. Procedures and Data Collection

A first face-to-face interview followed by a telephone interview at least 7 days later were conducted to obtain the study information described below.

#### 2.2.1. Socio-Demographic and Anthropometric Information

The following variables about children were collected in the first interview by means of a general questionnaire: place and date of birth, sex, academic level of parents or caregivers (elementary or less/secondary/university/higher education), place of residence, family income level, lifestyle, activity patterns, and sedentary behaviors. Height and weight data were declared by parents or caregivers, based on the child’s pediatric health card. Body mass index (BMI) was calculated and World Health Organization (WHO) reference standards were used to calculate BMI-Z scores [16,17]. BMI for age Z score was used to categorize children as ‘severe underweight’ (Z-BMI/age ≤ −3 standard deviation (SD)), ‘underweight’ (Z-BMI/age −3 to −2 SD), ‘normal BMI’ (Z-BMI/age −2 to +2 SD), or ‘overweight’ (Z-BMI/age +2 SD to +3 SD and ‘obese’ > +3 SD). Length/height for age Z score was used to categorize children as ‘low height’ (Z-height/age < −2 SD), ‘normal height’ (Z-height/age −2 to +2 SD), or ‘high stature’ (Z-height/age > +2 SD).

#### 2.2.2. Physical Activity and Sedentary Behavior Questionnaire

Physical activity and sedentary behaviors were assessed by a modification of a questionnaire previously validated in Colombian children <10 years old which was based on recall of activities carried out in a seven-day period [18]. Activities carried out by the child in one day (24 h) over the last week (a seven-day record) were documented, including information on hours of sleep and screen time; data for weekdays and weekend days were recorded separately. For more detailed information, see Madrigal et al. [15,19].

### 2.3. Procedures, Dietary Survey and Data Collection

Parents or caregivers helped completing two independent 24-h DR to determine children’s dietary intake (proxy report); comprising a week and a weekend day (non-consecutive), one being face-to-face and the other by telephone after ≥7 days. A detailed description of dietary intake including ingredients, and methods of preparation was structured as mealtimes (breakfast, mid-morning, lunch, mid-afternoon, dinner, and other moments) to calculate energy and nutrient distribution throughout the day.

In the present work, the EFSA recommendations to calculate misreporting was followed; the protocol proposed by EFSA is based mainly on the Goldberg [20] and Black [21] work. This method evaluates the reported energy intake (EIrep) against the presumed energy requirements. EIrep is expressed as a multiple of the mean basal metabolic rate estimated (BMRest) (from formulas), and it is compared with the presumed energy expenditure of the studied population. Then the ratio EIrep:BMRest is referred to as the physical activity levels (PAL) [22]. In addition, the protocol indicates that analyses should be done at group level and individually. Th first determines the overall bias to the reported EI, and the second shows the rate of under and over reporters. In the present article, we calculated the BMRest using the Schöfield equations [23]. The study of food and nutrient consumption encompasses methodological difficulties, especially when it comes to assessing it in a children cohort. Misreporting of the EsNuPI participants was previously assessed and reported by Madrigal et al. [21], where in the SRS, 84.7% (*n* = 598) were classified as plausible reporters and 15.3% (*n* = 108) non-plausible reporters (6.1% under—and 9.2% over-reporters). Regarding the age group, there were more under-reporters among the 6–<10 years group (7.6%), whereas the youngest (1 < 3 years) showed more over-reporters (20.5%). On the other hand, 83.5% (*n* = 618) of the AMS was classified as plausible energy reporters and 16.4% (*n* = 122) as non-plausible reporters (5.9% under- and 10.5% over-reporters). According to the age group, 10.8% (*n* = 20) were identified as under-reporters in the 6–<10 years old, while 14.7% (*n* = 43) were over-reporters in 1 < 3 years segment. The results included in this article were not adjusted for misreporting, because in the previous work from Madrigal et al. [21] the exclusion of misreporters resulted in no significant differences in the total energy intake (TEI) and the distributions of relative macronutrient intakes. Therefore, we might assume that it does not significantly modify the results and conclusions of this study.

Support materials as the “Tables of common home measures and habitual portion sizes for Spain population” [24,25] and the “Photo guide of common portions sizes of Spanish foods” [26] were used by interviewers for the adequate completion of the survey. In addition, the “VD-FEN 2.1” software, a Dietary Evaluation Program developed by the Spanish Nutrition Foundation (FEN) [25], was used to calculate the food, beverage and energy and nutrient intakes. Reported intakes were compared with the EFSA [27] recommendations for evaluating the carbohydrates and fiber consumption. The different food items were categorized into the following 18 food groups: “milk and dairy products”, “cereals”, “meat and meat products”, “oils and fats”, “bakery and pastry”, “fruits”, “vegetables”, “sugars and sweets”, “ready to cook/eat”, “other dairy products”, “beverages”, “legumes”, “eggs”, “fish and shellfish”, “appetizers”, “cereal-based baby foods and supplements”, “nuts” and “sauces and condiments”.

### 2.4. Statistical Analysis

The reported 746 food items that resulted from collection of dietary intake information were categorized into 18 food groups and transformed into energy and nutrient data for analysis by several statistical methods. The statistical method developed by Nusser et al. [28] -also known as the Iowa State University (ISU) method- was applied in order to eliminate the day-to-day variation in food consumption [29] as the average intake obtained using a 24 h DR for only a small number of days (observed intakes) does not satisfactorily represent the usual intake. The ISU method was applied using the PC-SIDE software (version 1.0, 2003, Iowa State University, Ames, IA, USA), designed for this purpose, which estimated the remain above or below the dietary reference cut-off values.

The Kolmogorov-Smirnoff normality test was used to determine the normality of the distribution of the variables. Mean, SD, median and interquartile range (IQR) were used for continuous variables and frequencies and percentages for categorical variables, to describe total carbohydrates (starch and total sugars) and fiber intake by type of cohort (SRS and AMS) gender and age groups. Comparisons by gender and age group between SRS and AMS were completed by Mann-Whitney U-test. Kruskal–Wallis Test and the Dunn to adjust for multiple comparison and adjustment of the p value with Bonferroni correction were used to calculate differences among each age group within cohorts. Level of significance was established at *p* < 0.05. Data analyses were performed using IBM SPSS 24.0 (IBM Corp., Armonk, NY, USA)

## 3. Results

A total of 1448 children between the ages of 1 to <10 years (49.7% girls and 50.3% boys) completed the study (Table 1). Their anthropometric, sociodemographic and physical activity characteristics are presented in Table 1, where it can be observed that there were significant differences between the SRS and the AMS only in weight and height for total cohort and when categorized by gender. Specifically, we found that children consuming adapted milk reported significantly lower weight and height (*p* < 0.001) than their SRS counterparts, except for girl’s height which was higher.

### 3.1. Total Carbohydrates, Starch, Total Sugar and Fiber Usual Intake in Children

Total carbohydrates, as well as starch and total sugar intakes are described in Table 2 for each cohort, by gender and age. When compared, we only observed significant differences for starch intakes amongst boys aged 1 to <3 years, where the AMS had a lower starch consumption (*p* ≤ 0.05), and for total sugar consumption in girls aged 6 to <10 years, where a higher intake was observed amongst those from the AMS group (*p* ≤ 0.05). On the other hand, when results were analyzed by age-groups we observed, as expected, that total carbohydrate, starch, and total sugar intakes were significantly higher amongst older groups independently of type of milk consumed. One exception were girls from the reference group (SRS), who had similar total sugar intakes regardless of the age group they belonged to.

We also assessed the percentage of children by age and gender who met the Reference Intake (RI) range for total carbohydrates (45–60% of TEI), and found that in all groups more than 50% of individuals met the recommendations, except for girls aged 6 to <10 years from the reference cohort in which only 46.9% complied the RI by EFSA. In addition, it is worth underlining that boys and girls from early ages (1 to <3 years) consuming adapted milk, had a higher level of compliance with recommendations than those from the reference group (90.6% and 82.7%, respectively).

Table 3 shows the results obtained for total carbohydrates, starch, and total sugar as a percentage of TEI by gender and age. The contribution of total carbohydrates to TEI was significatively higher (*p* < 0.05) in boys and girls aged 1 to <3 years, consuming adapted milks than those from reference cohort. Likewise, we observed that children from 1 to <3 years and 3 to <6 years age-groups consuming adapted milk had a significantly lower starch contribution to TEI (*p* < 0.05) than their counterparts. On the other hand, it is worth mentioning that, regardless of the type of milk consumed, boys aged 1 to <3 years had a higher carbohydrate and total sugar contribution to TEI, than those from older age segments. However, this age-group also showed a lower contribution from starch than the rest. Data shows the same tendency in girls for starch and total sugar contribution to TEI. Nevertheless, total carbohydrate contribution to TEI from girls do not change with age, regardless of type of milk consumed.

Table 4 displays the usual fiber intakes for each cohort, segmented by gender and age. Significant differences were only found between age groups 1 to 3 years and 4 to 6 years of children consuming adapted milk, since those from the elder group consumed higher fiber quantities than those from younger ages (*p* ≤ 0.05). It is also remarkable that as children grow older, regardless of gender and type of milk they consume, they are not able to meet the fiber AI specified by EFSA.

### 3.2. Contribution of Food and Beverage Groups to Total Carbohydrates, Starch, Total Sugar and Fiber Reported Intakes

**Figure 2:**
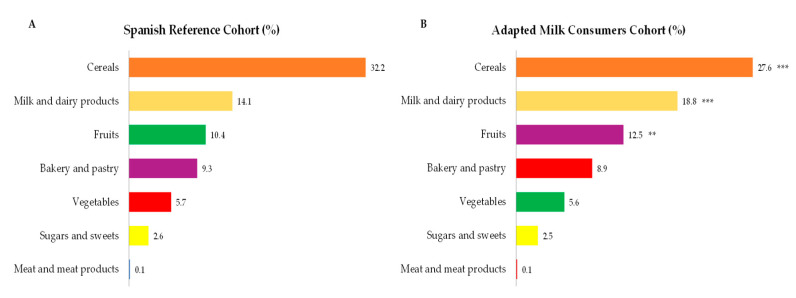
Dietary food and beverage groups contributing to total carbohydrate intakes (%) from the EsNuPI study population (“Spanish Pediatric Population”) in both Spanish Reference Cohort (**A**) and the Adapted Milk Consumers Cohort (**B**). ** *p* ≤ 0.01 compared to reference cohort (Mann-Whitney test). *** *p* ≤ 0.001 compared to reference cohort (Mann-Whitney test). Only foods contributing ≥0.1% to total carbohydrates intakes of the population have been included.

Main sources of total carbohydrate intakes amongst children from the EsNuPI study are presented in Figure 2, where cereals were the main contributors followed by milk and dairy products, fruits, bakery and pastry, vegetables and sugars and sweets. It is worth highlighting that in Figure 2, only those foods which contribute at least 0.1% to total carbohydrates intakes of the population have been included. In turn, we observed that cereals contributed in a lower proportion to total carbohydrate intakes from children consuming adapted milk (Figure 2B), where milk and dairy products (*p* ≤ 0.001) and fruits (*p* ≤ 0.001) accounted for a higher proportion when compared to carbohydrate intakes from children from the reference sample (Figure 2A).

**Figure 3:**
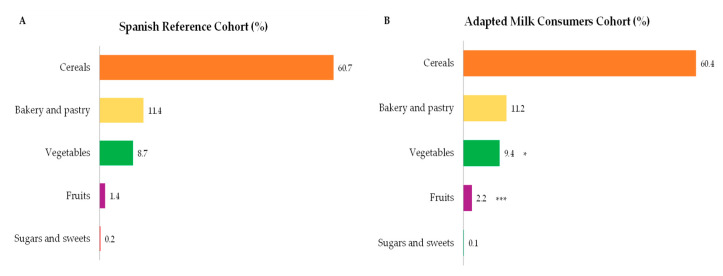
Dietary food and beverage groups contributing to total starch intakes (%) from the EsNuPI study population (“Spanish Pediatric Population”) in both reference cohort (**A**) and the Adapted Milk Consumers Cohort (**B**). * *p* ≤ 0.05 compared to reference cohort (Mann-Whitney test). *** *p* ≤ 0.001 compared to reference cohort (Mann-Whitney test). Only foods contributing ≥0.1% to total carbohydrates intakes of the population have been included.

The highest contributors to starch intakes within the studied population were cereals, bakery and pastry, vegetables, and fruits (Figure 3A,B). It is worth highlighting that in Figure 3, only those foods which contribute at least 0.1% to total starch intakes of the population have been included. A higher contribution to children’s starch intake was observed from the vegetable (*p* ≤ 0.05) and fruit group (*p* ≤ 0.001) amongst children consuming adapted milk compared to the reference cohort.

**Figure 4:**
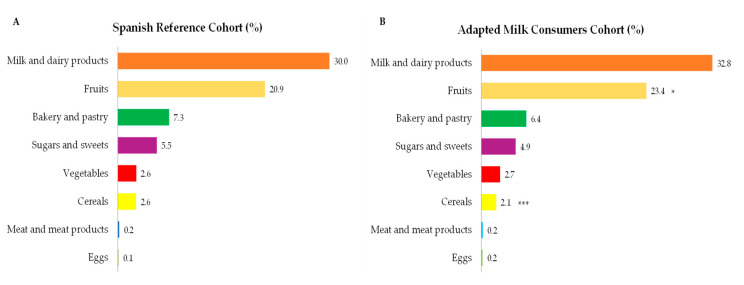
Dietary food and beverage groups contributing to total sugar intakes (%) from the EsNuPI study population (“Spanish Pediatric Population”) in both Spanish Reference Cohort (**A**) and the Adapted Milk Consumers Cohort (**B**). * *p* ≤ 0.05 compared to reference cohort (Mann-Whitney test). *** *p* ≤ 0.001 compared to reference cohort (Mann-Whitney test). Only foods contributing ≥0.1% to total carbohydrates intakes of the population have been included.

Regarding sources of total sugar intakes (Figure 4A,B), we found that major contributing food groups were milk and dairy products, fruits, bakery and pastry, sugars and sweets, vegetables, and cereals. In addition, while fruits showed a higher contribution to total sugar intakes from children consuming adapted milks compared to reference cohort (*p* ≤ 0.05), cereals accounted for a major proportion as a source of sugar in the latter (*p* ≤ 0.001). It is worth highlighting that in Figure 4, only those foods which contribute at least 0.1% to total sugar intakes of the population have been included.

**Figure 5:**
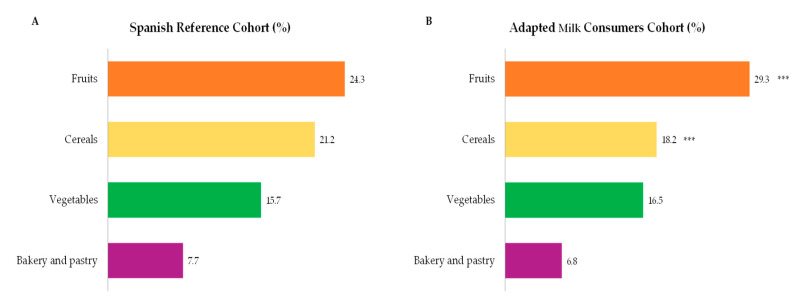
Dietary food and beverage groups contributing to total fiber intakes (%) from the EsNuPI study population (“Spanish Pediatric Population”) in both the Spanish Reference Cohort (**A**) and the Adapted Milk Consumers Sample (**B**). *** *p* ≤ 0.001 compared to reference cohort (Mann-Whitney test). Only foods contributing ≥0.1% to total carbohydrates intakes of the population have been included.

Finally, food groups with a higher contribution to fiber intakes were fruits, cereals, vegetables and bakery and pastry (Figure 5A,B). It is worth highlighting that in Figure 5, only those foods which contribute at least 0.1% to total fiber intakes of the population have been included. Fruits were significatively higher amongst children consuming adapted milks than in the reference cohort (*p* ≤ 0.001). However, cereals showed a higher contribution to fiber intakes in the Spanish Reference Cohort than in the Adapted Milk Consumers Cohort (*p* ≤ 0.001).

### 3.3. Total Carbohydrates, Starch, Total Sugar, and Fiber Intake in Children by Nielsen Geographical Area, Body Composition, and Income Level

Table 5 compiles results for total carbohydrates, starch, total sugars, and fiber from our study population segmented by Nielsen geographical areas inhabited by participants. We found that in the Spanish East zone, intakes across children from the AMS were significantly lower than average intakes from the reference group. The same tendency was observed for total carbohydrate intakes from children from the AMS living in the Center zone compared to those from the reference group. Again, starch intakes were higher amongst children from the reference group than those from the AMS living in Central and Northwest areas. Nonetheless, individuals from the AMS living in Northcentral areas had a significantly higher total sugar intakes than their SRS counterparts. Subsequently, we studied total carbohydrate, starch, total sugar, and fiber intakes in relation to body composition (Table 6). We observed that total carbohydrate intakes were significantly lower in children from the AMS in the range of normal weight and amongst those who were obese. In addition, children from the SRS presenting severe underweight consumed a lower quantity of total carbohydrates than those who presented normal weight. Regarding starch consumption, we found that in the AMS, independent of their body composition, children consumed lower starch quantities than their SRS counterparts, being these intakes significantly lower in children who were normal weight, overweight and obese (*p* < 0.001). Likewise, we observed that starch intakes amongst children who were obese was significantly reduced when compared to intakes from children with malnutrition and normal weight (*p* ≤ 0.05). Fiber intakes only showed a significant reduced intake amongst children consuming adapted milks who had a normal weight when compared to their counterparts (*p* < 0.001). Lastly, we found that total sugar intakes did not show any influence in body composition amongst the analyzed groups, as regardless of cohorts studied and body composition, total sugar intakes ranged from 70.3 (52.8–82.8) g/day in the AMS to 86.5 (41.7–111.9) g/day in the SRS, both in the severe underweight group, and there were no significant differences amongst all groups. Table 7 presents total carbohydrate, starch, total sugar, and fiber intakes by children from different income levels families. We observed that those who consumed adapted milk showed significantly lower carbohydrate intakes than their equals belonging to the middle (1501 to 2000 €) and high (>2000 €) income level (*p* < 0.001 and *p* < 0.05, respectively). Starch intakes presented a significant reduction amongst the AMS compared to their counterparts for middle and high family incomes (*p* < 0.001 and *p* < 0.001, respectively). Finally, we only observed a lower intake of fiber when compared to reference cohort, within the family groups who declared middle income levels (*p* < 0.01). On the other hand, when we compared total carbohydrates, starch, total sugar, and fiber intakes within the same cohort, we found that children from the SRS had higher intakes when they belonged to the highest level of family income. However, in the AMS, the group showing significantly lowest starch intakes, was the one belonging to the middle income compared to the rest of groups.

## 4. Discussion

The EsNuPI study represents the most recent and unique survey conducted in Spanish children aged one to <10 years, that delivers comprehensive information about specific nutrient intake and food consumption from a representative cohort of all kinds of milk consumers and from adapted milk consumers.

### 4.1. Total Carbohydrate Intakes and Sources

Carbohydrates are essential macronutrients for obtention of energy and main providers of glucose to body cells and brain in this stage of rapid development and growth [31]. Our findings indicated that the adequacy of carbohydrate intakes amongst children aged one to <10 years to EFSA recommendations was achieved by at least 50% of surveyed participants. But this also implies that about half the cohort participants were not meeting Reference Intakes Ranges (45–60% TEI) [7], leaving plenty of room for improvement in their dietary patterns.

When comparing the present results with previous studies in Spanish children, in the enKid [32] (*n* = 3534, 2–24 years), about 22 years ago (1998–2000) carbohydrate intakes from children were described as an average (P25–P75) of 245.6 ± 50.7 g/day (206.8–279 g/day, 42.8 ± 4.8% TEI) for boys and 200 ± 38.6 g/day for girls (175.2–223.9 g/day, 42.6 ± 5.1% TEI). These results showed that children were not even achieving the lower range level of 45% TEI recommended by EFSA.

In 2013, results from the ENALIA study [9] were expressed as percentage of the Acceptable Macronutrient Distribution Range (AMDR) that evaluated the distribution of participants relative to the total energy intake percentage. Median values for total carbohydrates were 46.8% (P10–P90) (41.2–52.3%) of total energy. These values show an increase from the ones observed in the EnKid study. The usual median proportion of energy intake from total carbohydrates was higher for infants from 6 to 12 months than for the other age groups. The proportion of participants with usual % TEI from carbohydrates below the lower limit of the AMDR was between 35.7–28.7% for boys, and 42.9–29.7% for girls. Children 4–8 years old showed the highest percentages under the AMDR. It must be considered that the ENALIA study was carried out during a period of economic crisis, which is known to be an important modifying factor for the dietary intake of families. In the ANIBES study, although only children from 9–12 years (*n* = 213) were included in the total cohort [8,10], their results describe an average total carbohydrate intake of 43.4–44.4% TEI for boys and girls, respectively. Noteworthy, higher total carbohydrate consumption was observed in younger age groups as compared to adults and older adults. Börnhorst, et al. assessed information from 8611 European children, from eight countries, aged 2 to 9 years, also by using 24 h DR and found that total carbohydrates accounted for 51.2% of TEI for all groups which is higher than our observed results [33].

In 2010, the EFSA published its Scientific Opinion on Dietary Reference Values for carbohydrates and dietary fiber, where data from dietary surveys for average carbohydrate intakes across European children and adolescents from 19 countries ranged between 43 to 58 TEI % [7]. Highest mean intakes were observed in the Czech Republic and Norway, while the lowest were found in Greece and Spain [7].

Trends occurring in Spain, according to the Food Consumption Survey evolution in the last decades, show that the percentage contribution of total carbohydrates has progressively decreased since the 1960s. Furthermore, in that decade, the energy profile was in line with recommendations [34]. However, it is relevant to mention the difficulty of establishing comparisons between results from studies on dietary intake because of the differences in dietary assessment methods, food composition tables employed, study population, age-categories selected and statistical estimation procedures.

Major total carbohydrate sources consumed amongst children in the current study were cereals (SRS vs. AMS: 32.2–27.6%), milk and dairy products (14.1–18.8%) and bakery and pastry (9.3–8.9%) Main food group contributors or sources of total carbohydrates amongst the total Spanish population were described in the ANIBES study [8] being grains (49%) and milk and dairy products (10%), in a higher and lower proportion when compared to our results, respectively. It must be noted that in the ANIBES study food group classification “bakery and pastry” were a subgroup of the group “grains” or “cereals” which is a methodological difference from our study and should be considered for comparisons. In addition, the type of carbohydrates provided by cereals (mainly starch), bakery and pastry (mainly starch and added sugars), and milk and dairy products (mainly lactose) differed widely, which may have an impact on health issues.

### 4.2. Starch Intakes and Sources

Daily starch consumption from our cohort ranged from 54.6–54.0 g/day for boys and girls aged 1 to <3 years (SRS, the group with higher intakes) to 107.5–99.2 g/day for boys and girls aged 6 to <10 years. These figures represented between 18.0–17.8% and 24.6–24.8% of TEI for those same age groups. However, in the ANIBES study [35], starch intakes amongst children 9 to 12 years were shown to be higher: 124.3 ± 37.1 g/day for boys and 119.9 ± 35.3 g/day for girls, which accounted for a mean contribution to TEI of 24.6 ± 4.3% and 25.4 ± 5.2%, for boys and girls respectively.

The main dietary starch suppliers from the EsNuPI’s participants were cereals (SRS vs. AMS: 60.7–60.4%), bakery and pastry (11.4–11.2%), vegetables (8.7–9.4%) and fruits (1.4–2.2). Interestingly, a higher contribution to children’s starch intake was observed from the vegetable and fruit groups amongst children consuming adapted milk compared to the reference cohort. Starch is the major carbohydrate of the cereal group composition. It is worth remarking that the second main contributor to starch intakes were the bakery and pastry group, which consumption is controversial, particularly amongst children because of the high reported contents of fats and free and added sugars these foods usually provide. These are highly palatable and accepted products amongst youngsters, but their excessive consumption concerns nutrition and public health professionals as not only these products are characterized for being very energy dense and having “empty calories”, but also because they may displace the consumption of healthier choices (i.e., fruits, nuts, etc.) that provide essential micronutrients [12,36].

### 4.3. Total Sugar Intakes and Sources

Our results showed that total sugar intakes amongst children ranged from a median of 77.9–75.9 g/day in boys and girls aged 1 to <3 years to 86.1–73.4 g/day in boys and girls aged 6 to <10 years (Spanish Reference Cohort). In the Adapted Milk Consumers Cohort intakes were 72.3–67.9 g/day in boys and girls aged 1 to <3 years, up to 84.3–86.6 g/day in boys and girls aged 6 to <10 years. We also found that total sugar intakes were significantly higher amongst older groups regardless of the type of milk they consumed, except for girls from the reference cohort, who had similar total sugar intakes irrespective of the age group they belonged to. Total sugar intakes accounted for a level higher than the WHO recommendation of limiting daily free sugar intakes to less than 10% [12]. In the ANIBES study [35], total sugar mean daily intake were 93.7 ± 35.3 g/day in boys and 88.4 ± 30.1 g/day in girls aged 9 to 12 years, which were higher than our results. These intakes were also shown to be higher in children and adolescents than in adults and older adults, evidencing the relevance of this dietary component at younger stages of life. The review by Azaïs-Braesco, et al. [13] showed that across different European countries, Belgium and The Netherlands reported higher total sugar consumption amongst children of both genres.

In the present study main contributors to total sugar intakes were milk and dairy products and fruits which were higher than the contributions described in the ANIBES study [8] for milk and dairy products and fruits, although figures are presented for the overall population (9–75 years). In this regard, we can suggest that total sugar sources from children were from food groups where the highest sugar proportion is intrinsic. Remarkably, the ANIBES showed an important percentage of total sugar derived from non-alcoholic beverages (19%), sugars and sweets (15%) and grains (12%), that in or study accounted for less than half of these proportions. However, we have to bear in mind age ranges from ANIBES accounted for older children and therefore their dietary patterns might be considerably different from those of the EsNuPI. Moreover, it should be taken into account that, opposite to non-alcoholic beverages, which contain added sugars e.g., sucrose and glucose and fructose syrups, milk and dairy mainly supply lactose, as well as fruits provides fructose, both naturally-occurring sugars which undoubtedly would have differential metabolic and health impacts in infancy. When studying other European countries, main sources of total sugar were sweet products and beverages [13].

### 4.4. Dietary Fiber Intakes and Sources

The EFSA recommendations on dietary fiber daily intakes for children (AI) are 10 g for children 1 to 3 years, 14 g for those 4 to 6 years and 16 g for age range of 7 to 10 years [7]. Our results showed that daily fiber intake compliance with recommendations were higher amongst younger ages as in the segment of children aged 1–3 years, 61–67.2% of participants showed intakes >AI (SRS and AMS), while in the group of 7 to 10 years only 7.2–17.9% achieved the recommended levels. Absolute daily fiber intakes ranged from a median of 10 (8–14) g/day in boys 1–3 years from AMS and girls of the same age from SRS, to 12 (9–16) g/day in boys aged 4–6 years from the SRS. This fact brings to attention that this beneficial dietary pattern might progressively decrease with age, by substitution with other foods that are less rich in fiber and more appealing to older infants, who are also presented with broader food choices. Previous studies like the ENALIA study [9] reported higher mean fiber intakes across all studied age-groups, starting with 12.6 ± 3.6 g/day for boys and 11.8 ± 3.8 for girls aged 1–3 years, and 15.7 ± 2.7 g/day for boys and 15.3 ± 3.1 g/day for girls aged 4–8 years. But again, in the ANIBES study [8], mean daily intakes were lower: 11.5 ± 4.0 g/day amongst boys and 12.2 ± 4.6 g/day in girls aged 9 to 12 years. These two studies did not provide data, however, on the proportion of individuals meeting the EFSA requirements.

A 2107 review by Alison et al. [37] studied the fiber intakes of European children, assessing data from 11 surveys and large studies including children 1–4 years and 20 surveys for children 4–12 years. For the youngest age group (1–4 years), dietary fiber intakes ranged from 8 to 12 g/day. For children aged 4–12 years, the range in intakes were from 10 to 18 g/day for boys and 8 to 18 g/day for girls, showing an increase of intakes with age which we also found in our results. Authors also note that intakes were very similar from country to country, with lower values in Ireland and the UK. However, proportions of these populations complying with recommendations were not provided.

The present study showed that major fiber sources were fruits, cereals, and vegetables. In contrast, in the ANIBES study [8] grains were the main sources for children aged 9–12 years, followed by vegetables and fruits. The fact that fruits are the main fiber source reflects a positive pattern within the cohort habits. The SENC guidelines [38,39] recommend at least 3 serving per day that most Spanish children do not comply with [9]. Fruit intake amongst younger populations include further benefits as enhanced vitamin and mineral intake, and other dietary bioactive compounds of interest.

The ANIBES study [8] found that amongst the main fiber sources from the grain group, bread represented the highest contributor for children aged 9–12 years. No further characteristics on bread composition were provided, while it is well known that bread ingredients define its fiber content (whole flour vs. white or refined flour). In the aforementioned review from European countries [37] grains were also the major source of fiber; fruit made a greater contribution to fiber for children than for adults, with values ranging from below 10% for older children in the UK to 26% for children in Spain, the latter being comparable to the ones obtained in the present study. The Spanish study mentioned is from Serra-Majem, et al. [40] conducted in Catalonia, Evaluation of Nutritional Status in Catalonia (ENCAT) and with children aged 10 to 17 years which might explain the higher cereal contribution to fiber intakes.

### 4.5. Body Composition, Socioeconomic and Geographical Factors

Our results from the current EsNuPI assessment revealed no specific overall pattern related to nutrient intake and body composition. However, relevant findings were the fact that children from the SRS presenting severe underweight consumed a lower quantity of total carbohydrates than those who presented normal weight. Fiber intakes only showed a significant reduced intake amongst children consuming adapted milks who had a normal weight when compared to their counterparts, and interestingly total sugar intakes did not show any influence in body composition amongst the analyzed groups.

In the Identification and Prevention of Dietary—and Lifestyle-Induced Health Effects in Children and Infants (IDEFICS) study, children aged 2–9 years from eight European countries were recruited in 2007–2008 [41,42]. Their results showed that that children of parents with lower socioeconomic status (SES) might be at higher risk of unhealthy eating, as researchers found a strong inverse association of SES with a processed food consumption pattern. Healthy food policies should target on informing and empowering families from lower SES on healthy eating to exert a significant impact on the nutritional status of infants [41]. In the present study, we assessed children’s total carbohydrates, starch, total sugar, and fiber intakes in relation to family income to find that no clear pattern was evident for lower SES and nutrient consumption. Only when comparing within the same sample group, we found that children from the reference sample had higher intakes of all assessed nutrients, except for total sugar, when they belonged to the highest level of family income. In this regard, results from the EnKid study also showed that children from a lower socioeconomic background and those whose mothers had a lower level of education had a higher consumption of sweets and bakery products as well as sugary and salted snacks, but lower vegetable intakes [43].

### 4.6. Strengths and Limitations

The EsNuPI study encompassed a representative cohort of the Spanish children population and is an updated source of information on dietary intakes and patterns and socioeconomic factors. The main strength of this study was the possibility of assessing a representative cohort of the Spanish children aged one to <10 years living in urban areas and the possibility of comparing the results of this reference population with a sample of adapted milk consumers of the same age. Furthermore, the 24-h DR information was collected following the methodology recommended by EFSA (The PAN CAKE-Pilot study) [44].

A limitation of this study is that young children are less able to remember, estimate, and cooperate in dietary assessment procedures than older children, so information should be obtained from parents or caregivers [45], taking into consideration the bias that might represent for instance, meals taken at school by children and the under- or overreporting derived from parents own beliefs related to diet and health. The potential recall bias introduced by parents in reporting their children’s diets is associated with a tendency to overestimate foods accepted as healthy and underestimate the least healthy options. The assessment of misreporting performed by Madrigal et al. [19] showed that the exclusion of under or over reporters has no significant influence in children’s TEI, therefore they were included in the present study.

Limiting added sugar intakes in accordance to the WHO guidelines [12] to less than 10% is a strong recommendation, that gains importance moreover amongst the children population; but it is expressed as “free sugars” (those that are added, plus those from honey, syrup or fruit juices), therefore, comparison to our results is limited since we obtained “total sugars” for the different food and beverage groups. In the present study, we did not differentiate added sugar from total sugar intakes, the main reason being Food Composition Tables and Databases we used lacked the specific data regarding added (i.e., sucrose) or intrinsic sugars (i.e., lactose). Furthermore, European regulation for labelling of food products [46] do not mandate the quantification and declaration of added sugars on labels, (where only “Carbohydrates” and “of which sugars” can be found, but this “sugar” content contemplates added as well as intrinsic quantities. Consumers may only identify that sugar is actually added to a product by reading the ingredients list, where it is mandatory to declare. In a recently published work conducted on processed products from the Spanish market [47], sugar contents from labels were compared to analytically obtained values; however, no assessment of added sugar contents was possible as these are chemically identical to intrinsic ones, underlining the need from manufacturer’s data on these ingredients.

## 5. Conclusions

According to our results, although a high proportion of children showed total carbohydrates intakes in line with recommendations by public health authorities, still a significant number presented insufficient total carbohydrate and fiber intakes, while total sugar consumption was high. Nutrition education efforts should be encouraged to improve diets and life quality of future generations, especially in this life stage where healthy patterns are learnt and assimilated, as well to encourage adequate reformulation policies. Public health strategies targeted at early age are key to improve overall population’s health and should be aimed at families and school environments where children develop. 

## Figures and Tables

**Table 1:** Personal, anthropometric, and socioeconomic data by gender and age group in the Spanish Pediatric Population (EsNuPI) study. Adapted from [30].

		Spanish Reference Cohort (SRS)			Adapted Milk Consumers Cohort (AMS)		
		Total	Boys	Girls	Total	Boys	Girls
Total population, *n* (%)	*n*	707 (48.8%)	357 (50.5%)	350 (49.5)	741 (51.2%)	371 (50.1%)	370 (49.9%)
Age group, *n* (%)	1 to <3 years	162 (22.9%)	84 (23.5%)	78 (22.3%)	294 (39.7%)	144 (38.8%)	150 (40.5%)
	3 to <6 years	244 (34.5%)	122 (34.2%)	122 (34.9%)	262 (35.4%)	128 (34.5%)	134 (36.2%)
	6 to <10 years	301 (42.6%)	151 (42.3%)	150 (42.9%)	185 (25%)	99 (26.7%)	86 (23.2%)
Anthropometric characteristics, median (interquartile range)	Weight (kg)	19.0 (14.6–26.0)	19.0 (15.0–26.6)	19.0 (14.0–26.0)	15.0 *** (12.0–20.0)	16.0 *** (12.0–22.0)	15.0 *** (12.0–20.0)
	Height (cm)	110.0 (95.0–126.0)	110.0 (95.0–127.0)	110.0 (94.0–126.0)	98.0 *** (86.0–115.0)	98.0 *** (87.0–118.0)	98.0 *** (95.0–110.0)
	BMI (kg/m^2^)	16.5 (15.2–18.0)	16.5 (15.3–18.0)	16.5 (15.1–18.0)	16.3 (15.1–18.0)	16.4 (15.3–17.9)	16.3 (14.9–18.0)
	Z-BMI/Age	0.6 (−0.3–1.5)	0.6 (−0.3–1.5)	0.6 (−0.3–1.4)	0.5 (−0.3–1.4)	0.45 (−0.3–1.4)	0.5 (−0.3–1.4)
	Z-Weight/Age	0.5 (−0.3–1.2)	0.4 (−0.4–1.2)	0.6 (−0.3–1.3)	0.6 (−0.3–1.4)	0.6 (−0.1–1.4)	0.5 (−0.3–1.4)
	Z-Height/Age	−0.3 (−1.2–0.9)	−0.2 (−1.1–1.0)	−0.4 (−1.3–0.7)	−0.4 ** (−1.4–0.6)	−0.4 ** (−1.4–0.6)	−0.4 (−1.5–0.6)
Physical Activity Level (PAL), median (interquartile range)	1 to <3 years	1.6 (1.3–1.8)	1.6 (1.4–1.8)	1.5 (1.3–1.8)	1.5 (1.3–1.7)	1.5 (1.3–1.8)	1.5 (1.3–1.7)
	3 to <6 years	1.6 (1.4–1.7)	1.6 (1.4–1.7)	1.5 (1.4–1.7)	1.5 (1.4–1.7)	1.5 (1.4–1.7)	1.5 (1.4–1.7)
	6 to <10 years	1.6 (1.4–1.7)	1.6 (1.4–1.8)	1.6 (1.5–1.7)	1.6 (1.5–1.7)	1.6 (1.5–1.8)	1.6 (1.5–1.7)
Size of the municipality, *n* (%)	50.001 a 300.000 people	376 (53.2%)	193 (54.1%)	183 (52.3%)	406 (54.8%)	204 (55.0%)	202 (54.6%)
	>300.000 inhabitants	331 (46.8%)	164 (45.9%)	167 (47.7%)	335 (45.2%)	167 (45.0%)	168 (45.4%)
Highest level of education achieved by one of the parents, *n* (%)	≤10 years of education	23 (3.3%)	10 (2.9%)	13 (3.8%)	14 (1.9%)	7 (1.9%)	7 (1.9%)
	Secondary education	416 (60.5%)	219 (62.9%)	197 (57.9%)	414 (57.0%)	208 (57.5%)	206 (56.6%)
	University studies	249 (36.2%)	119 (34.2%)	130 (38.2%)	298 (41.0%)	147 (40.6%)	151 (41.5%)
Income Level (€/month), *n* (%)	Low (<1500 €)	171 (24.2%)	79 (22.1%)	92 (26.3%)	163 (22.0%)	84 (22.6%)	79 (21.4%)
	Medium (1501 to 2000 €)	126 (17.8%)	67 (18.8)	59 (16.9%)	134 (18.1%)	64 (17.3%)	70 (18.9%)
	High (>2000 €)	226 (32.0%)	123 (34.5%)	103 (29.4%)	238 (32.1%)	110 (29.6%)	128 (34.6)
	No answer/Does not know	184 (26.0%)	88 (24.6%)	96 (27.4%)	206 (27.8%)	113 (30.5%)	93 (25.1%)
Number of feeding bottles or glasses of milk per day, *n* (%)	Less than 2	222 (32.9%)	110 (32.0%)	115 (33.8%)	178 (24.1%)	92 (24.9%)	86 (23.3%)
	2 or more	459 (67.1%)	234 (68.0%)	225 (66.2%)	561 (75.9%)	278 (75.1%)	283 (76.7%)

BMI: Body Mass Index; PAL: Physical Activity Level. The PAL was calculated for individual and group level according to the European Food Safety Authority (EFSA) protocol to assess misreporting [22]. Values are presented as median (interquartile range) or percentage per group. ** *p* < 0.01 difference vs. reference cohort (Mann-Whitney’s U test). *** *p* < 0.001 difference vs. reference cohort (Mann-Whitney’s U test).

**Table 2:** Total carbohydrates, starch and total sugar usual intakes and prevalence of adequacy for carbohydrate intake (percentage of population between 45–60% RI) by gender and age group in the Spanish Pediatric Population (EsNuPI) study.

	Group	Group by Age	RI (%)	Boys				Girls	
				*n*	Median (P25–P75)	% between 45–60% RI	*n*	Median (P25–P75)	% between 45–60% RI
Carbohydrates (g/day)	SRS	1 to <3 years	45–60	84	136.3 (108.4–172.9) ^a^	60.6	78	134.7 (112.3–166.3) ^a^	62.6
		3 to <6 years	45–60	122	166.2 (145.3–191.2) ^b^	58.1	122	158.1 (132.2–194.5) ^b^	51.6
		6 to <10 years	45–60	151	194.3 (150.3–227.7) ^c^	67.1	150	176.7 (143.2–217.5) ^b^	46.9
	AMS	1 to <3 years	45–60	144	137.7 (112.8–167.1) ^a^	90.6	150	138.2 (115.2–166.2) ^a^	82.7
		3 to <6 years	45–60	128	166.1 (138.5–199.9) ^b^	57.2	134	170.6 (141.1–197.8) ^b^	63.3
		6 to <10 years	45–60	99	183.5 (149.6–215.4) ^b^	64.9	86	180.0 (148.0–209.4) ^b^	69.9
Starch (g/day)	SRS	1 to <3 years	-	84	54.6 (34.7–79.2) ^a^	-	78	54.0 (32.8–78.5) ^a^	-
		3 to <6 years	-	122	86.0 (65.1–109.4) ^b^	-	122	77.9 (60.5–107.0) ^b^	-
		6 to <10 years	-	151	107.5 (76.4–132.1) ^c^	-	150	99.2 (75.0–127.1) ^c^	-
	AMS	1 to <3 years	-	144	42.1 (28.5–66.1) *^,a^	-	150	47.3 (34.1–70.0) ^a^	-
		3 to <6 years	-	128	77.4 (63.2–102.3) ^b^	-	134	79.4 (62.9–99.7) ^b^	-
		6 to <10 years	-	99	93.5 (73.3–121.6) ^c^	-	86	95.3 (73.6–108.1) ^b^	-
Total sugar (g/day)	SRS	1 to <3 years	-	84	77.9 (61.5–89.8) ^a^	-	78	75.9 (57.5–91.6)	-
		3 to <6 years	-	122	78.0 (60.9–97.2) ^a,b^	-	122	75.5 (64.5–94.0)	-
		6 to <10 years	-	151	86.1 (67.5–106.6) ^b^	-	150	73.4 (58.4–91.3)	-
	AMS	1 to <3 years	-	144	72.3 (55.7–91.4) ^a^	-	150	67.9 (56.5–84.0) ^a^	-
		3 to <6 years	-	128	80.8 (64.7–95.8) ^a,b^	-	134	78.4 (64.3–95.6) ^b^	-
		6 to <10 years	-	99	84.3 (68.8–103.2) ^b^	-	86	86.6 (64.1–103.2) *^,b^	-

Reference intake range (RI) [7], Spanish Reference Cohort (SRS) and Adapted Milk Consumers Cohort (AMS). Values are presented as median (interquartile range) or percentage per group. Values that do not share superscript are significantly different between age-groups for each gender and in each cohort type (*p* ≤ 0.05; Kruskal–Wallis test and the Dunn test to adjust for multiple comparison and adjust the p-value with Bonferroni correction) and asterisk indicate statistically significant difference between cohort type difference *vs* reference cohort (*p* ≤ 0.05; Mann-Whitney’s U test)).

**Table 3:** Total carbohydrates, starch, and total sugar distribution (% of total energy) in the Spanish Pediatric Population (EsNuPI) study in both Spanish Reference Cohort, Adapted Milk Consumers Cohort.

	Group	Group by Age	Boys		Girls	
			*n*	Median (P25–P75)	*n*	Median (P25–P75)
Carbohydrates (%)	SRS	1 to <3 years	84	46.4 (41.8–50.7) ^a^	78	45.9 (39.9–51.3)
		3 to <6 years	122	44.9 (41.1–49.5) ^b^	122	44.4 (39.3–49.4)
		6 to <10 years	151	45.0 (41.2–48.6) ^b^	150	45.4 (40.6–49.5)
	AMS	1 to <3 years	144	48.4 (43.6–52.5) *^,a^	150	47.6 (43.6–53.0) *
		3 to <6 years	128	46.2 (41.6–50.1) ^b^	134	46.0 (41.3–50.0)
		6 to <10 years	99	45.0 (41.2–49.3) ^b^	86	45.8 (41.9–49.2)
Starch (%)	SRS	1 to <3 years	84	16.4 (13.6–23.6) ^a^	78	17.8 (13.7–22.9) ^a^
		3 to <6 years	122	24.0 (19.0–28.1) ^b^	122	22.5 (18.1–26.6) ^b^
		6 to <10 years	151	24.7 (20.9–28.6) ^b^	150	24.8 (20.7–29.7) ^c^
	AMS	1 to <3 years	144	15.0 (10.6–20.0) *^,a^	150	17.3 (12.7–22.3) ^a^
		3 to <6 years	128	21.8 (18.7–25.5) *^,b^	134	21.8 (17.6–26.5) ^b^
		6 to <10 years	99	23.2 (19.2–27.7) ^b^	86	23.1 (19.7–27.3) ^b^
Total sugar (%)	SRS	1 to <3 years	84	25.7 (20.5–31.1) ^a^	78	25.5 (20.6–30.6) ^a^
		3 to <6 years	122	20.8 (17.5–24.6) ^b^	122	21.8 (18.3–25.4) ^b^
		6 to <10 years	151	20.1 (16.9–24.7) ^b^	150	19.3 (15.9–22.7) ^c^
	AMS	1 to <3 years	144	25.4 (21.3–30.8) ^a^	150	24.4 (19.8–29.4) ^a^
		3 to <6 years	128	22.9 (19.1–25.8) ^b^	134	21.7 (18.4–24.6) ^b^
		6 to <10 years	99	21.1 (17.8–24.0) ^b^	86	22.0 (17.3–25.0) ^b^

Spanish Reference Cohort (SRS), Adapted Milk Consumers Cohort (AMS) and Confidence interval (CI). Values are presented as mean (confidence interval), median (interquartile range) and percentage per group. * *p* < 0.05 difference vs. reference cohort (Mann-Whitney’s U test). Values that do not share superscript are significantly different between age-groups for each gender and in each cohort type (*p* ≤ 0.05; Kruskal–Wallis test and the Dunn test to adjust for multiple comparison and adjust the *p*-value with Bonferroni correction).

**Table 4:** Fiber usual intake and prevalence of adequacy (percentage of population >AI) by gender and age group in the Spanish Pediatric Population (EsNuPI) study.

	Group	Group by Age	AI (g/Day)	Boys			Girls		
				*n*	Median (P25–P75)	>AI (%)	*n*	Median (P25–P75)	>AI (%)
Fiber (g/day)	SRS	1–3 years	10	135	11.3 (7.9–14.2)	61.0	127	10.2 (7.7–13.9)	67.2
		4–6 years	14	105	12.0 (8.6–15.5)	45.1	106	11.2 (7.8–14.9)	34.8
		7–10 years	16	117	12.1 (8.6–15.2)	17.9	117	11.3 (9.0–14.3)	11.3
	AMS	1–3 years	10	200	10.4 (7.5–13.7) ^a^	65.3	214	10.1 (7.8–13.4)	65.2
		4–6 years	14	94	11.9 (9.2–14.7) ^b^	38.1	92	10.9 (8.5–13.9)	20.4
		7–10 years	16	77	11.0 (8.4–14.3) ^a,b^	0.0	64	9.6 (8.0–14.2)	7.2

Adequate intakes (AI) [7], Spanish Reference Cohort (SRS) and Adapted Milk Consumers Cohort (AMS). Values are presented as median (interquartile range) and percentage per group Values that do not share superscript are significantly different between age—groups for each gender and in each cohort type (*p* ≤ 0.05; Kruskal-Wallis test and the Dunn test to adjust for multiple comparison and adjust the *p*-value with Bonferroni correction).

**Table 5:** Total carbohydrates, starch, total sugar, and fiber intakes by geographical distribution amongst the Spanish Reference Cohort (SRS) and the Adapted Milk Consumers Cohort (AMS) in the Spanish Pediatric Population (EsNuPI) study.

Geographical Distribution Group (Nielsen Areas)	*n*		Carbohydrates (g/Day)			Starch (g/Day)		Total Sugar (g/Day)		Fiber (g/Day)	
	SRS	AMS	SRS	AMS	SRS		AMS	SRS	AMS	SRS	AMS
Barcelona (Metropolitan Area)	55	61	151.5 (141.0–191.7)	155.8 (134.9–185.0)	88.9 (68.9–107.3)		79.8 (61.5–105.9)	69.7 (57.9–84.5)	68.5 (58.3–87.7)	11.5 (8.4–15.2)	11.0 (8.9–14.2)
Canary Islands	29	42	1656.0 (136.9–201.2)	153.8 (128.1–185.8)	67.5 (55.2–107.9)		68.8 (46.5–91.8)	84.2 (77.4–106.4)	78.1 (58.6–96.3)	11.2 (9.7–14.4)	9.7 * (7.0–13.3)
Center	44	77	177.2 (146.1–202.4)	160.9 * (129.8–183.2)	83.7 (65.6–105.2)		67.6 ** (42.1–86.1)	84.4 (71.2–104.0)	82.0 (67.7–98.7)	9.9 (7.8–13.4)	9.7 (7.2–13.4)
East	111	80	204.6 (156.5–232.9)	167.6 *** (133.9–200.7)	114.0 (78.1–139.1)		86.9 *** (53.3–112.8)	82.3 (62.6–100.2)	69.5 ** (56.5–87.4)	11.5 (9.9–15.0)	9.9 ** (7.7–13.6)
Madrid (Metropolitan Area)	130	152	153.2 (128.3–183.8)	150.2 (118.9–174.8)	74.6 (52.3–96.6)		68.2 * (39.0–91.3)	74.4 (60.3–91.9)	74.7 (57.7–89.3)	10.8 (7.8–14.1)	11.1 (8.1–14.0)
Northeast	57	74	145.9 (122.3–189.1)	142.1 (116.6–173.7)	79.2 (62.3–114.9)		60.7 ** (41.1–93.3)	67.6 (52.4–81.8)	68.7 (54.7–82.5)	12.2 (9.1–16.3)	11.1 (8.8–13.3)
Northwest	94	86	153.3 (123.7–190.4)	149.5 (125.8–184.5)	72.5 (49.3–108.5)		62.9 * (41.7–85.5)	77.1 (58.8–96.5)	77.3 (61.5–95.5)	11.5 (7.1–14.9)	10.4 (8.1–13.8)
North Central	68	62	178.3 (147.3–240.9)	196.4 (153.1–264.7)	89.6 (72.3–136.0)		90.3 (56.2–130.8)	82.4 (68.8–106.9)	90.9 * (77.4–131.1)	13.1 (10.4–17.5)	13.2 (10.1–15.9)
South	119	107	158.3 (123.9–194.2)	168.7 (132.4–198.4)	77.5 (48.8–108.1)		71.2 (45.2–93.8)	80.5 (62.2–98.8)	86.2 (66.0–105.8)	9.7 (6.9–13.2)	10.3 (7.8–13.4)

Values are presented as median (interquartile range) per group. * *p* < 0.05 difference vs. reference cohort (Mann-Whitney’s U test). ** *p* < 0.01 difference *vs* reference cohort (Mann-Whitney’s U test). *** *p* < 0.001 difference *vs* reference cohort (Mann-Whitney’s U test).

**Table 6:** Total carbohydrates, starch, total sugar, and fiber intake in relation to body composition amongst the Spanish Pediatric Population (EsNuPI) study in the Spanish Reference Cohort (SRS) and the Adapted Milk Consumers Cohort (AMS).

		*n*		Carbohydrates (g/Day)		Starch (g/Day)		Total Sugar (g/Day)		Fiber (g/Day)	
Group		SRS	AMS	SRS	AMS	SRS	AMS	SRS	AMS	SRS	AMS
BMI-for-age percentile	Severe underweight	11	17	135.6 ^a^ (109.8–257.1)	130.1 (108.3–165.9)	65.3 (39.1–145.2)	53.8 ^a,b^ (25.8–85.7)	86.5 (41.7–111.9)	70.3 (52.8–82.8)	12.1 (10.1–15.7)	9.7 (7.6–14.6)
	Underweight	80	83	125.4 ^a,b^ (125.4–184.1)	158.3 (130.0–197.1)	76.4 (52.8–104.5)	74.6 ^b^ (53.0–99.5)	74.9 (58.3–96.9)	75.0 (63.2–89.9)	10.4 (7.4–13.2)	10.8 (8.0–14.1)
	Normal	359	391	167.23 ^b^ (139.3–204.7)	159.6 * (128.0–193.4)	84.5 (63.2–113.1)	75.3 ***^,^^b^ (47.4–97.8)	78.0 (62.6–93.8)	75.2 (58.8–93.0)	11.8 (8.5–14.9)	10.5 *** (8.0–13.8)
	Overweight	153	133	165.9 ^a,b^ (127.0–203.7)	165.5 (132.7–197.6)	84.6 (54.6–116.8)	67.7 ***^,^^a,b^ (40.0–92.4)	76.0 (59.3–98.5)	84.5 (65.0–99.9)	11.3 (7.5–14.5)	11.4 (8.7–14.1)
	Obesity	104	117	165.6 ^a,b^ (135.6–206.9)	153.7 * (126.2–182.7)	86.4 (63.5–112.2)	62.0 ***^,^^a^ (37.5–89.6)	80.1 (65.5–95.3)	79.4 (61.9 – 94.0)	11.0 (8.5–14.9)	10.8 (7.7–13.6)

Values are presented as median (interquartile range) per group. * *p* < 0.05 difference *vs* reference cohort (Mann-Whitney’s U test). *** *p* < 0.001 difference *vs* reference cohort (Mann-Whitney’s U test). Values that do not share superscript are significantly different between age-groups for each gender and in each cohort type (*p* ≤ 0.05; Kruskal–Wallis test and the Dunn test to adjust for multiple comparison and adjust the *p*-value with Bonferroni correction).

**Table 7:** Total carbohydrates, starch, total sugar, and fiber intake by monthly income levels from the Spanish Pediatric Population (EsNuPI) study in the Spanish Reference Cohort (SRS) and the Adapted Milk Consumers Cohort (AMS).

		n		Carbohydrates (g/Day)		Starch (g/Day)		Total Sugar (g/Day)		Fiber (g/Day)	
Group		SRS	AMS	SRS	AMS	SRS	AMS	SRS	AMS	SRS	AMS
Income Level (€/month)	Low (<1500 €)	171	163	150.9 ^a^ (123.3–189.9)	157.7 (128.9–187.7)	77.2 ^a^ (50.0–106.6)	74.5 ^a^ (54.1–98.2)	76.9 (61.5–91.3)	76.0 (57.9–92.6)	10.7 ^a^ (7.4–14.3)	9.6 ^a^ (7.7–13.2)
	Medium (1501 to 2000 €)	126	134	173.5 ^b^ (148.8–215.5)	153.9 *** (122.5–178.9)	89.7 ^b^ (66.6–117.5)	59.7 ***^,^^b^ (42.0–84.6)	84.3 (62.8–108.1)	74.6 (61.3–94.5)	11.2 ^a,b^ (8.5–14.2)	9.7 **^,^^a^ (7.4–12.6)
	High (>2000 €)	226	238	177.8 ^b^ (136.8–217.9)	162.8 * (129.1–201.4)	93.4 ^b^ (65.0–130.3)	75.1 ***^,^^a^ (44.3–103.1)	78.5 (60.0–94.6)	79.3 (61.5–93.6)	11.8 ^b^ (8.8–15.2)	11.4 ^b^ (8.5–14.1)
	No answer/ doesn’t know	184	206	158.8 ^a^ (132.2–188.8)	159.6 (127.9–191.1)	81.1 ^a^ (56.1–104.2)	73.2 *^,^^a,b^ (42.7–96.1)	75.4 (62.1–92.8)	77.7 (63.6–96.5)	11.5 ^a,b^ (7.8–14.4)	11.2 ^b^ (8.6–14.5)

Values are presented as median (interquartile range) per group. * *p* < 0.05 difference *vs* reference cohort (Mann-Whitney’s U test), ** *p* < 0.01 difference *vs* reference cohort (Mann-Whitney’s U test), *** *p* < 0.001 difference *vs* reference cohort (Mann-Whitney’s U test). Values that do not share superscript are significantly different between age-groups for each gender and in each cohort type (*p* ≤ 0.05 Kruskal–Wallis test and the Dunn test to adjust for multiple comparison and adjust the *p*-value with Bonferroni correction).
